# Apparent Dead Ends: Emerging Therapeutic Pathways in Treatment-Resistant Depression

**DOI:** 10.1192/j.eurpsy.2026.11409

**Published:** 2026-07-31

**Authors:** M. Calvo-Valcárcel, M. A. Andreo-Vidal, C. Rodríguez -Valbuena, A. Monllor-Lazarraga, B. Rodriguez-Rodriguez, O. Martín-Santiago, A. San Román-Uria, J. C. Fiorini-Talavera

**Affiliations:** Psychiatry, Hospital Clínico Universitario de Valladolid, Valladolid, Spain

## Abstract

**Introduction:**

Depressive disorders are the leading cause of disability worldwide. We speak of treatment-resistant depression (TRD) when a case of major depressive disorder (MDD) fails to respond adequately to at least two different antidepressants, given at the right dose and duration. Over the years, different therapeutic strategies have been used: combining medications, augmentation, switching treatments, as well as electroconvulsive therapy (ECT). Despite these options, it is estimated that around 30% of patients with MDD are resistant to treatment. More recently, intranasal esketamine has been approved as a novel treatment for this group of patients.

**Objectives:**

An update on current approaches to treatment-resistant depression.

**Methods:**

We report the case of a 65-year-old female with a long-standing affective disorder, beginning over ten years ago following the loss of two pregnancies. She is admitted privately to a Convalescence Hospital and subsequently requires psychiatric hospitalization for an acute exacerbation of anxiety-depressive symptoms, during which she is treated with venlafaxine, amisulpride, trazodone, and diazepam. After discharge, she is referred to a structured Day Treatment Program. Despite this, her condition deteriorates, presenting with pronounced psychomotor inhibition, severe anxiety, and functional impairment, prompting readmission. Pharmacological adjustments includes dose escalation of amitriptyline and augmentation with mirtazapine. Due to limited response, electroconvulsive therapy (ECT) is initiated, producing partial improvement. Following this, intranasal esketamine is introduced and is currently maintained as part of her therapeutic regimen.

**Results:**

The patient exhibits a long-standing, treatment-resistant depressive disorder. Initial pharmacotherapy with venlafaxine, amisulpride, trazodone, and diazepam yields minimal improvement, with persistent anxiety, psychomotor inhibition, and functional impairment. Subsequent dose escalation of amitriptyline and augmentation with mirtazapine provides partial symptom relief. Electroconvulsive therapy (ECT) produces limited clinical improvement. Following this partial response, intranasal esketamine is initiated as maintenance therapy, resulting in mood stabilization, reduction of anxiety, and progressive functional recovery. No significant adverse events are observed during esketamine administration.

**Conclusions:**

Intranasal esketamine constitutes a novel therapeutic modality for the management of treatment-resistant depression, exhibiting potential to stabilize mood and enhance functional outcomes in patients demonstrating inadequate response to conventional pharmacotherapy. Furthermore, other innovative approaches, including neuromodulation via transcranial magnetic stimulation (TMS) and electroconvulsive therapy (ECT), continue to represent pertinent strategies in the comprehensive management of this patient population.

**Disclosure of Interest:**

None Declared

